# Exploring Intervention Strategies for Microbial Biofilms in the Food Industry Based on a Biomolecular Mechanism Perspective: Recent Advances and Emerging Trends

**DOI:** 10.3390/foods14244192

**Published:** 2025-12-06

**Authors:** Luchuanyang Sun, Bingbing Xu, Ye Tao, Yan Liang, Xianggui Chen

**Affiliations:** 1 School of Food and Bio-Engineering, Xihua University, Chengdu 610039, China; bettyxu121@163.com; 2 School of Food and Pharmacy, Zhejiang Ocean University, Zhoushan 316022, China; taoye@zjou.edu.cn (Y.T.); liangyan@zjou.edu.cn (Y.L.); 3 Food Microbiology Key Laboratory of Sichuan Province, Chengdu 610039, China

**Keywords:** biofilm, extracellular polymeric substance, food processing environments, persister cells, quorum sensing, sanitation strategies

## Abstract

Microbial biofilms in food processing environments pose significant challenges due to their exceptional resistance to conventional sanitation methods, presenting substantial risks to food safety and public health. This review systematically evaluates recent advances in understanding biofilm development across key stages, i.e., initial microbial adhesion, extracellular polymeric substance production, biofilm maturation including resistant phenotypes such as persister cells, and dispersion. Particular emphasis is placed on the molecular mechanisms underlying biofilm formation and the regulatory roles of cyclic-di-GMP and quorum sensing. Crucially, we highlight emerging targeted interventions including enzyme-mediated extracellular polymeric substance disruption, microenvironmental manipulation, quorum sensing inhibitors, metabolic reactivation of persisters (“wake-and-kill”), and controlled biofilm dispersion techniques, clearly outlining their practical applicability and potential limitations in real-world food industry contexts. Moreover, this review uniquely integrates innovative technological developments such as responsive antimicrobial coatings, real-time biosensors, predictive modeling systems, and precision biotechnology approaches. Uniquely, this review integrates molecular mechanisms with practical, stage-specific sanitation strategies and provides actionable insights that can enhance biofilm control, contributing to safer food production practices and im-proved public health outcomes.

## 1. Introduction

**Figure 1:**
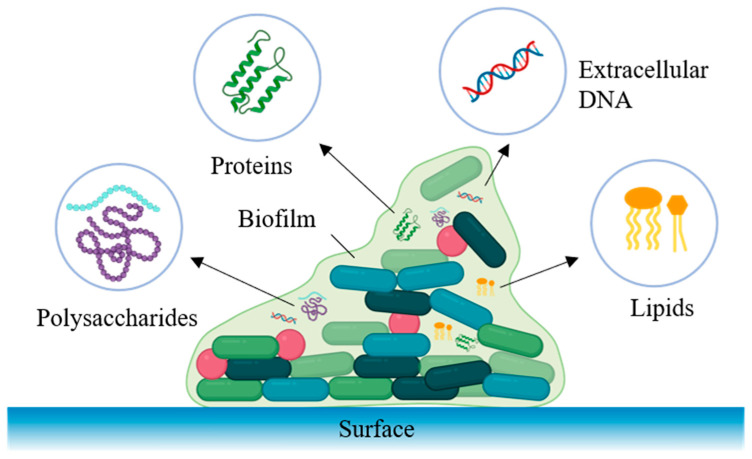
Schematic representation of the key regulatory compounds in biofilm.

Biofilm contamination remains one of the most persistent threats in food processing environments, significantly compromising food hygiene and public health. And is estimated to cause substantial economic losses through product recalls, equipment downtime, and sanitation costs in dairy, meat, and ready-to-eat sectors. As illustrated in Figure 1, biofilms are structured microbial communities embedded in self-produced extracellular polymeric substances (EPS), primarily composed of polysaccharides, proteins, extracellular DNA (eDNA), and lipids [1]. These biofilm-associated microbes exhibit significantly enhanced resistance compared to their planktonic counterparts, tolerating conventional sanitation methods, antimicrobials, and environmental stresses, thus complicating eradication efforts and posing severe public health risks [2].

Biofilm formation progresses sequentially through distinct and highly regulated stages, including initial reversible adhesion governed primarily by physicochemical forces (e.g., electrostatic interactions, van der Waals forces, hydrophobic effects) and bacterial appendages (flagella, pili) [3]. Each developmental stage involves specialized molecular and regulatory mechanisms orchestrated by intracellular signaling pathways, notably cyclic-di-GMP (c-di-GMP) and quorum sensing (QS), enabling bacteria to finely adjust biofilm behavior to environmental cues and community conditions [4,5].

Conventional sanitation practices in food industries, predominantly based on chemical disinfectants and physical removal methods, often fail to penetrate or eliminate mature biofilms fully, leaving behind persistent and resistant subpopulations [6]. Resistant persister cells surviving within biofilms frequently trigger recontamination after cleaning, highlighting critical limitations in current industrial sanitation strategies. These persistent contamination problems underscore an urgent need for targeted, mechanism-based interventions, as inadequate biofilm control directly contributes to high-risk outbreaks and costly recalls associated with pathogens such as *Listeria monocytogenes* and *Salmonella* spp. in global food production.

Recent research has begun to address these gaps by exploring innovative, stage-specific biofilm control methods. Biofilm control can be aligned with developmental stages: surface engineering limits initial adhesion; enzymatic and biosurfactant treatments disrupt EPS; microenvironmental shifts increase antimicrobial susceptibility; QS inhibitors and “wake-and-kill” strategies target persisters; and controlled dispersion followed by sanitation prevents secondary contamination [7,8,9]. Furthermore, emerging advanced technologies, such as responsive antimicrobial surfaces, real-time biosensors for early biofilm detection, predictive biofilm growth modeling, and precision biotechnology approaches including CRISPR-based antimicrobials, hold significant promise for revolutionizing biofilm management in food processing environments. However, several critical research gaps remain. Multi-species biofilms prevalent in real food-processing environments are still poorly understood compared to single-species laboratory models. In addition, the influence of diverse industrial surface materials on biofilm physiology and resistance has not yet been systematically characterized. Importantly, although CRISPR- and phage-based interventions show strong potential, their large-scale implementation in food plants faces regulatory, stability, and safety challenges that warrant further investigation.

This review provides a comprehensive and systematic assessment of current molecular insights into biofilm formation and maturation, emphasizing recent advancements in targeted intervention methods across all developmental stages. The unique contribution of this review lies in its integration of diverse and innovative control strategies, explicitly evaluating their practical applicability, benefits, and limitations within real-world food industry contexts. By synthesizing multidisciplinary approaches into actionable guidelines, this review aims to facilitate more effective biofilm control practices, thereby significantly enhancing food safety management and protecting public health.

## 2. Initial Adhesion-Targeting Strategies in Food Chain

### 2.1. Physicochemical Forces Governing Initial Adhesion

Initial attachment to food-contact surfaces is governed by a balance of weak physicochemical forces and bacterial appendage-mediated interactions. In aqueous environments at neutral pH, both bacterial cells and common processing materials such as stainless steel and glass generally exhibit net negative surface charges [10,11]. This creates electrostatic double-layer repulsion that forms an energy barrier during approach [12]. At closer distances, however, universal van der Waals forces and hydrophobic interactions (often described within extended DLVO theory) can outweigh this repulsion, enabling reversible adhesion [13]. The strength of these interactions is highly dependent on ionic strength, which compresses the electric double layer and reduces repulsion, thus, adhesion is inhibited at low ionic strength but favored at higher ionic strength when the Debye length shortens [12,13]. Divalent cations such as Ca^2+^ can further bridge negatively charged surfaces and reduce the energy barrier for contact [13].

Beyond abiotic interactions, bacterial surface appendages actively promote attachment. Flagella can extend beyond the electrostatic barrier, contacting and adsorbing onto surfaces, an effect supported by studies showing reduced adhesion in *E. coli* lacking functional flagella [11,14]. Pili, particularly type IV pili (T4P), enable initial tethering and subsequent retraction, pulling the cell into close proximity to the substratum [15]. These appendages allow bacteria to overcome repulsive forces and position themselves within nanometer-scale distances where van der Waals, hydrophobic, or specific ligand–receptor interactions can secure attachment. In summary, initial adhesion arises from the interplay between surface physicochemistry and bacterial appendages. Although electrostatic repulsion tends to oppose attachment, it is counterbalanced by attractive van der Waals and hydrophobic forces, especially under elevated ionic strength, and by active bridging through flagella and pili [12,14]. These early events facilitate the transition to irreversible adhesion and subsequent biofilm development.

### 2.2. Strategies to Prevent Initial Adhesion

**Figure 2:**
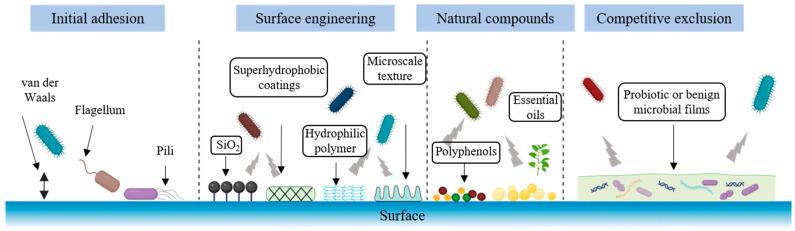
Strategies for preventing initial bacterial adhesion on food-contact surfaces.

Given the importance of early-stage attachment, numerous strategies have been developed to disrupt or prevent the initial adhesion of bacteria on food-contact surfaces. Figure 2 provides a conceptual overview linking these strategies to their modes of action, showing how surface engineering, natural anti-adhesion agents, and competitive microbial coatings function as the primary defense to block biofilm initiation in food-processing environments.

#### 2.2.1. Surface Engineering and Material Modification

One effective strategy is to modify the physicochemical properties of the surface to make it less amenable to bacterial adhesion. By altering surface charge, energy, roughness or wettability, the goal is to create an anti-fouling interface. Coating stainless steel with a nanoscale layer of silica has been shown to significantly reduce the initial attachment of *Listeria monocytogenes* (*L. monocytogenes*). The silica-modified steel becomes more hydrophobic (water contact angle ~115° vs. 56° for uncoated steel), which in turn reduces the real contact area between bacterial inoculum and the surface [16]. In practical terms, fewer bacteria can find anchoring points on such a coated surface, leading to lower cell adhesion counts [16]. Empirically, silica-coated steel exhibited decreased *Listeria* adherence and facilitated easier removal of cells during rinsing, without inhibiting bacterial growth (indicating an anti-adhesive rather than bactericidal effect) [16]. Other surface modifications under investigation include creating superhydrophobic coatings that trap air and minimize bacterial contact, grafting hydrophilic polymer brushes that generate a hydration layer repelling cells, and imparting microscale texture that reduces effective attachment area. Each of these engineering approaches aims to raise the energetic barrier for adhesion or otherwise minimize the favorable interactions between bacteria and the substratum. By rendering the surface anti-adhesive, one can delay or prevent biofilm formation at its inception.

#### 2.2.2. Natural Anti-Adhesion Compounds

A second strategy leverages naturally derived compounds, such as plant polyphenols and essential oils (EOs), to inhibit bacterial attachment. Many plant polyphenols possess anti-biofilm properties that include interference with initial surface adhesion. For instance, certain tannins and flavonoids can adsorb to either the bacterial surface or the contact material and hinder the physicochemical interactions required for adhesion [17]. Notably, 7-epiclusianone (a polyphenol from *Garcinia* species) and tannic acid have been reported to prevent the surface attachment of bacteria in vitro [17]. These molecules may alter bacterial cell surface hydrophobicity or block adhesin proteins, thereby reducing the ability of cells to attach. Similarly, EOs from herbs and spices have shown strong anti-adhesion effects at sub-inhibitory concentrations. EOs components such as eugenol (from clove), thymol (thyme), carvacrol (oregano), and cinnamaldehyde (cinnamon) can disrupt early biofilm establishment. In a recent study, a citrus EOs dramatically reduced Bacillus cereus biofilm adhesion on stainless steel. The oil-treated surface had only ~33% of the bacterial attachment compared to an untreated surface [18]. The mechanism often involves EOs and their phenolic constituents weakening the bacterial membrane or downregulating genes involved in adhesion, thereby inhibiting the initial sticking of cells [19]. As these natural compounds are generally recognized as safe in foods, they offer a promising green approach. Surfaces or equipment can be coated or treated with plant extracts/EOs to continuously deter microbial attachment in food processing environments. Ongoing research is optimizing the delivery of such compounds (e.g., incorporating them into edible films or polymer coatings) to achieve long-lasting anti-adhesion effects.

#### 2.2.3. Competitive Exclusion by Probiotic or Benign Microbial Films

Another approach to preventing pathogenic biofilm formation is to pre-colonize surfaces with harmless bacteria that exclude the pathogens. In this strategy, often termed microbial competitive exclusion or biological antagonism, a non-pathogenic strain (for example, lactic acid bacteria commonly used as probiotics) is encouraged to form a thin biofilm on the surface, occupying binding sites and resources so that incoming pathogens cannot easily attach. There is strong evidence that probiotic biofilms can serve as a living barrier against undesirable microbes. Several studies in food industry settings have shown that introducing *Lactobacillus* or *Bacillus* probiotics onto equipment surfaces can prevent the attachment of *Listeria, Salmonella,* and other spoilage or pathogenic bacteria [20]. The protective biofilm outcompetes invaders by competing for binding sites (steric hindrance) and nutrients, and sometimes by secreting antagonistic substances (e.g., bacteriocins, biosurfactants) that inhibit pathogen growth. A probiotic *Lactobacillus* biofilm on stainless steel was reported to significantly reduce *Salmonella enteritidis* (*S. enteritidis*) adhesion in a factory environment, simply by occupying the surface and creating unfavorable local conditions for the pathogen. By actively excluding pathogens at the initial adhesion phase, probiotic coatings can halt biofilm problems before they start. This strategy is attractive because it employs naturally occurring food-grade microbes instead of chemicals and can be self-sustaining once the beneficial biofilm is established. Current research in this area focuses on identifying robust probiotic strains that adhere to industrial surfaces and tolerate cleaning regimens, as well as understanding how multi-species interactions on surfaces can be harnessed to keep equipment pathogen-free [20].

Overall, strategies like surface property optimization, natural anti-adhesion compounds, and beneficial microbial films not only mitigate early biofilm formation, but also differ significantly in cost-effectiveness: natural plant extracts and probiotic coatings offer more economical and sustainable options for routine use, whereas engineered nano-coatings provide high performance but currently involve higher material and manufacturing costs for large facilities. Despite extensive progress in surface engineering, the specific ways in which different industrial surface materials, such as stainless steel, polymers, and food-grade rubber, shape early adhesion and subsequent biofilm development remain insufficiently understood, representing a key gap in current research. However, once attachment occurs, bacteria rapidly begin producing EPS, shifting the challenge from preventing adhesion to disrupting matrix formation.

## 3. Targeting Biofilm Formation and Maturation in Food Industry Settings

### 3.1. Composition and Function of the Biofilm EPS Matrix

Once bacteria irreversibly attach to the surface, they initiate the synthesis and secretion of substantial amounts of EPS, creating a three-dimensional matrix that binds the microbial community together [21]. EPS typically consists of polysaccharides, proteins, eDNA, and lipids, although specific compositions differ significantly among bacterial species [22]. Biofilms of *Salmonella enterica* (*S. enterica*) and *E. coli* contain amyloid fibers (curli proteins) and cellulose, providing structural stability through a dense, fibrillar network [23]. Conversely, *Staphylococcus aureus* (*S. aureus*) biofilms rely heavily on poly-β-1,6-N-acetylglucosamine (PIA/PNAG) polysaccharides and abundant eDNA for structural support [24].

EPS components intertwine, forming a cohesive, viscoelastic matrix that serves as both an adhesive and protective barrier. eDNA, polysaccharides, and proteins collectively strengthen the EPS matrix and help biofilms remain firmly attached to food-contact surfaces [25,26]. eDNA contributes to early adhesion, while polysaccharides form a hydrated gel that retains nutrients and supports cell survival. In addition, matrix proteins, including structural fibers and enzymes, reinforce the biofilm architecture and aid in resource utilization. Moreover, lipids and bacterial membrane vesicles incorporated into EPS modulate their permeability and hydrophobic properties, thereby influencing biofilm resilience [27].

EPS synthesis is tightly regulated by intracellular signaling pathways, particularly the second messenger c-di-GMP [28]. Elevated c-di-GMP concentrations promote the transcription of EPS biosynthesis genes, thus driving matrix formation during the transition from planktonic to sessile lifestyle [29]. Additionally, QS systems regulate EPS production in response to population density. In species like *Pseudomonas aeruginosa* (*P. aeruginosa*), QS signals trigger matrix polymer synthesis at high cell densities, fortifying biofilm integrity [30]. Conversely, in *Vibrio cholerae* (*V. cholerae*), QS at high densities represses EPS production and facilitates biofilm dispersion [31].

### 3.2. Intervention Strategies Targeting EPS

**Figure 3:**
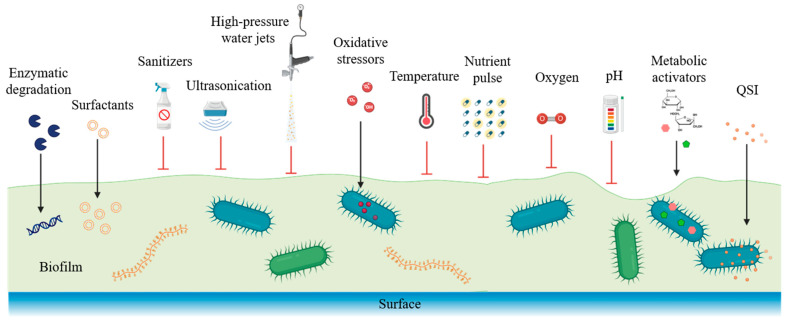
Intervention strategies targeting biofilm maturation, EPS disruption, microenvironment manipulation, and persister cell control.

Given that the EPS matrix significantly enhances biofilm resilience by acting as a protective barrier, strategies specifically targeting EPS are effective in biofilm control, particularly within food industry contexts. Figure 3 synthesizes how EPS disruption (via enzymes or biosurfactants), microenvironmental manipulation, and persister-targeted treatments form a combined intervention framework specifically adapted for food industry sanitation workflows, highlighting points where routine cleaning procedures can integrate targeted molecular strategies.

Enzymatic degradation constitutes one promising approach, employing enzymes such as DNase, dispersin B (glycoside hydrolase), and proteases to dismantle the EPS matrix structurally [32]. For example, DNase, which is readily obtainable from commercial suppliers, efficiently reduces biofilm biomass by cleaving eDNA, a critical component of the EPS in foodborne pathogens like *L. monocytogenes* on stainless steel surfaces, thereby significantly improving the effectiveness of subsequent sanitization [33]. Similarly, dispersin B, which remains an experimental enzyme not yet available for industrial-scale sanitation use, specifically targets poly-N-acetylglucosamine polysaccharides essential for staphylococcal biofilm structure, disrupting matrix cohesion and facilitating deeper sanitizer penetration [34]. Proteolytic enzymes such as proteinase K and the food-grade protease alcalase, both commercially available, have also shown substantial efficacy in degrading proteinaceous EPS components, particularly amyloid fibers produced by pathogens like *S. enterica* and *E. coli*, thereby further supporting sanitation efforts [35].

Additionally, EPS-disrupting biosurfactants such as commercially available rhamnolipids produced by *P. aeruginosa* can significantly destabilize biofilms by interfering with polysaccharide–protein interactions, thereby increasing susceptibility to antimicrobials [8]. Experimental studies using sub-inhibitory doses of rhamnolipids reported a substantial reduction in *Listeria* biofilm density on stainless steel surfaces, especially when used in combination with traditional sanitizers like peracetic acid [36,37]. Moreover, recent dairy-industry cleaning trials demonstrated that combining enzymatic pretreatment with oxidizing sanitizers achieved up to a three-log reduction in pathogen counts [38]. Physical disruption methods such as ultrasonication or high-pressure water jets combined with EPS-degrading enzymes have further amplified biofilm removal efficacy, significantly improving cleaning outcomes on industrial surfaces [39]. Recent investigations have also explored natural compounds, such as tea polyphenols and EOs derivatives like thymol and carvacrol, as alternative EPS-disrupting agents, demonstrating marked reductions in EPS production and biofilm biomass in foodborne pathogens [40]. Finally, quorum sensing inhibitors (QSIs), including halogenated furanones and flavonoids, have been effectively used to prevent EPS synthesis. Function in an early regulatory stage, blocking QS-mediated EPS gene expression before matrix accumulation occurs, thus reducing biofilm robustness and persister emergence [41].

Overall, integrating enzymatic, chemical, physical, natural compound-based, and QS inhibition approaches offers a robust and multifaceted strategy, with enzyme-assisted sanitation being the most immediately effective for mature EPS-rich biofilms in food plants, whereas biosurfactants and QSIs provide promising but currently more costly or regulatory-limited options for routine sanitation cycles.

### 3.3. Microenvironmental Effects on Biofilm Development and Stability

As biofilms mature, the accumulation of cells and EPS creates steep gradients of nutrients, oxygen, waste metabolites, and pH. These gradients result in distinct microenvironments within the biofilm, often structured into active, nutrient-rich outer regions and nutrient-deprived, hypoxic inner zones [42]. In mature *P. aeruginosa* biofilms, peripheral cells exhibit lower c-di-GMP levels, remaining motile and proliferative, whereas central cells display high c-di-GMP levels and increased EPS synthesis, entering dormancy to withstand nutrient limitations [3].

EPS acts as a buffer against external stresses, retaining water to prevent desiccation and neutralizing extreme pH fluctuations, thus maintaining biofilm homeostasis [43]. Nutrient availability significantly influences biofilm behavior. Severe nutrient deprivation often triggers cells to enter dormancy or increase EPS production, while sudden nutrient influx can stimulate dispersion as cells attempt to exploit new resources [44]. Additionally, QS molecules and environmental signals (oxygen depletion, acidic waste buildup) regulate gene expression profiles within localized biofilm regions, orchestrating adaptive responses or dispersion event [45].

### 3.4. Intervention via Microenvironment Manipulation

Targeting heterogeneous microenvironments within biofilms has emerged as an effective strategy for destabilizing resilient communities in food-processing environments. Controlled oxidative stressors such as low-dose hydrogen peroxide or peracetic acid can penetrate biofilms and disrupt internal homeostasis, leading to structural destabilization and enhanced susceptibility to antimicrobials [46,47]. Sublethal peracetic acid, for example, has been shown to disturb nutrient and redox gradients in *L. monocytogenes* and *S. enterica* biofilms, thereby sensitizing previously dormant cells to sanitation treatments [48]. Temperature-based interventions can further augment biofilm control. Mild heating increases EPS permeability, while rapid thermal shifts induce physiological stress that reactivates dormant cells and improves subsequent sanitizer penetration [49]. Another promising strategy is the nutrient-pulse method, in which brief exposure to sugars or organic acids (e.g., glucose, mannitol, lactic acid) stimulates metabolic activity in dormant cells [50]. Following such activation, antimicrobial treatments have shown markedly improved efficacy, with studies reporting substantial eradication of mature *P. aeruginosa* and *E. coli* biofilms when nutrient pulses precede disinfectant application [51]. Manipulating oxygen availability offers another route to destabilization. Short oxygen pulses trigger oxidative metabolic shifts that disrupt anaerobic zones, increasing overall vulnerability to sanitizers [52]. Controlled aeration has been shown to enhance disinfectant effectiveness against anaerobic *L. monocytogenes* biofilms [53]. Finally, pH modulation through brief acidification or alkalization can weaken EPS cohesion and alter biofilm architecture, improving antimicrobial penetration. Mild pH shifts have been demonstrated to compromise EPS integrity and enhance the activity of disinfectants used in routine food industry sanitation [53].

Overall, strategically manipulating biofilm microenvironments, through oxidative stress, temperature modulation, nutrient pulses, oxygenation, and pH adjustment, provides a comprehensive approach for dismantling mature biofilms and improving sanitation outcomes in food production systems.

### 3.5. Population Behavior and Phenotypic Changes During Biofilm Maturation

Upon entering the maturation stage, biofilm communities undergo a series of adaptive phenotypic changes, among which the emergence of persister cells is particularly noteworthy [54]. Persister cells constitute a small subpopulation characterized by a dormant metabolic state and exhibit extreme tolerance to antimicrobial agents. Notably, this resistance is not derived from genetic mutations but results from reversible phenotypic shifts in gene expression [55]. As biofilms age, nutrient depletion and accumulation of metabolic waste induce many cells to enter stationary or dormant phases to avoid death, thereby substantially increasing the proportion of persister cells. Studies [56] involving mature biofilms of *E. coli* and *P. aeruginosa* have observed that persister cells become significantly more abundant in mid-to-late logarithmic phases compared to early biofilm stages, often by several orders of magnitude. Due to their profoundly reduced metabolic activity, persister cells are largely unaffected by antibiotics and disinfectants that target active growth processes such as cell division and metabolism. Classic studies by Grassi et al. [57] have shown that persister cells are primary contributors to multidrug tolerance in biofilms, often surviving antimicrobial treatments and subsequently regenerating the biofilm population upon removal of stressors.

In addition to persister cells, mature biofilms can harbor other phenotypic variants, such as small colony variants (SCVs). These SCVs are characterized by altered metabolic pathways and modified gene expression profiles, which better equip them for survival in the challenging microenvironments within biofilms, thereby increasing community diversity and resilience [58].

Furthermore, complex QS-mediated behaviors play critical roles during biofilm maturation, orchestrating communal responses and structural adjustments. In high-density conditions, QS systems such as Las/Rhl in *P. aeruginosa* induce production of biosurfactants like rhamnolipids, creating hydrophobic channels that facilitate nutrient transport and waste expulsion [59]. QS signaling can also regulate secretion of enzymes responsible for EPS remodeling or controlled localized matrix degradation, influencing the timing of biofilm dispersal [60]. Conversely, certain species such as *V. cholerae* utilize QS signaling at high cell densities to suppress EPS synthesis, thus promoting dispersal and preventing overcrowding. Therefore, the role of QS during biofilm maturation can vary significantly, either reinforcing biofilm integrity (as observed in species like *E. coli* and *P. aeruginosa*) or promoting its breakdown (as seen in *V. cholerae*), depending on species-specific strategies and ecological contexts [61].

These adaptive phenotypic variations and coordinated community behaviors confer mature biofilms with remarkable resistance to conventional cleaning and sanitation methods. The combination of EPS barriers, metabolically inert persister cells, and coordinated stress responses collectively renders even aggressive antimicrobial treatments ineffective [62]. Mature biofilms thus resemble fortified structures that prioritize maintenance and defense, preparing for eventual dispersal and colonization of new niches. This phenomenon explains recurring contamination events observed in food processing environments, where pathogen populations often rebound shortly after sanitation efforts. The pathogens persist undetected within resilient mature biofilms in dormant states, quickly regrowing when cleaning concludes.

Consequently, special attention must be given during biofilm maturation to resistant subpopulations like persisters and to QS-mediated resistance and dispersion mechanisms. Developing targeted intervention strategies to specifically address these resistant phenotypes and community coordination pathways is therefore essential to effectively manage biofilm-associated risks in food production and processing environments.

### 3.6. Intervention Targeting Persisters and QS

Persister cells represent a critical challenge in biofilm control, as their metabolic dormancy confers exceptional tolerance to conventional antimicrobials. To effectively target these dormant subpopulations, recent studies have developed innovative “wake-and-kill” strategies, involving the controlled introduction of metabolic activators such as sugars (mannitol, glucose) or organic acids to stimulate persister cells into active growth. Once reactivated, these previously dormant cells become vulnerable to antimicrobial treatments. Study demonstrated that applying mannitol at sub-inhibitory concentrations effectively reactivated persisters within mature *P. aeruginosa* and *E. coli* biofilms, increasing their susceptibility to antibiotics by orders of magnitude and resulting in significant biofilm eradication [63]. Similarly, combinations of glucose or fructose pulses with standard antibiotics have successfully reduced persister populations within *L. monocytogenes* and *S. enterica* biofilms commonly found in food processing environments, demonstrating enhanced sanitation effectiveness compared to antimicrobials alone [64].

Another potent approach involves the disruption of QS, a bacterial communication mechanism crucial for coordinating collective behaviors such as EPS production, biofilm maturation, and persistence. QSIs, including plant-derived compounds such as halogenated furanones, flavonoids, and polyphenols, have demonstrated significant promise in biofilm control. These QSIs disrupt signaling pathways, thereby attenuating EPS synthesis, biofilm development, and persister formation. Recent studies revealed that halogenated furanones markedly inhibited QS-controlled EPS production in *P. aeruginosa* biofilms, substantially reducing their robustness and facilitating effective removal during subsequent sanitation procedures [65]. Furthermore, flavonoids extracted from natural sources such as tea or citrus peels have similarly shown potent QS inhibition properties, resulting in decreased biofilm biomass and reduced persister populations in pathogenic biofilms like those formed by *S. aureus* and *L. monocytogenes* [40]. Recent experiments combining QS inhibitors with conventional antimicrobials have further validated these approaches, showing that biofilms treated with QS inhibitors were significantly more susceptible to disinfectants, achieving biofilm reductions not attainable with disinfectants alone [66]. Additionally, combining QS inhibitors with enzymatic or surfactant-based treatments presents synergistic effects, substantially improving sanitation outcomes in food industry settings [67].

Collectively, employing “wake-and-kill” metabolic activation strategies alongside targeted QS inhibition forms a powerful, multifaceted intervention, with “wake-and-kill” being particularly promising for industrial adoption due to its low chemical input, strong sanitation synergy, and ease of integration into existing cleaning-in-place (CIP) systems. However, even with these targeted approaches, biofilms remain dynamic systems capable of dispersing when environmental cues shift. Understanding how and when dispersion occurs is therefore essential, as dispersed cells pose heightened contamination risks.

## 4. Biofilm Dispersion and Control Strategies

### 4.1. Mechanisms Triggering Biofilm Dispersion

Biofilm dispersion is the release of cells from a mature biofilm into a planktonic state, initiating contamination spread in food-processing environments. This transition is mainly regulated by intracellular signals such as reduced levels of c-di-GMP [42]. Elevated c-di-GMP concentrations generally promote bacterial attachment and matrix synthesis [68]. However, upon encountering unsuitable conditions for continued attachment, such as nutrient limitation, accumulation of metabolic wastes, or overcrowding, cells activate c-di-GMP phosphodiesterases (PDEs), rapidly degrading c-di-GMP and inducing profound phenotypic changes [69]. Such changes include suppression of EPS synthesis, degradation of the existing matrix, and reactivation of motility structures such as flagella, enabling cells to escape the biofilm and regain motility.

In addition to internal second messenger signaling, bacteria also sense external environmental and population-density cues to initiate dispersion. QS, for example, serves as a crucial dispersion trigger in certain species. *V. cholerae* represents a classic model where, at high cell densities, QS signals accumulate and induce expression of c-di-GMP PDEs, thereby suppressing *vps* polysaccharide gene expression, dismantling the matrix, and leading to coordinated community dispersion. In contrast, *S. aureus* employs the QS system Agr at the stationary growth phase to stimulate secretion of various hydrolytic enzymes, including proteases and nucleases, which systematically degrade the EPS matrix, facilitating cellular egress. These cases illustrate how different bacteria can coordinate biofilm dispersal through integrated QS and c-di-GMP signaling pathways once certain thresholds of density or environmental signals are reached.

NO is another important endogenous signal triggering dispersion. At nanomolar concentrations, NO is perceived by many bacteria as an indicator of hypoxia or environmental stress [70]. Studies with *P. aeruginosa* have demonstrated significantly impaired dispersion in mutants deficient in NO synthesis, whereas mutants accumulating excessive NO undergo premature biofilm disintegration [71]. Exogenous addition of NO donors at low concentrations rapidly induces biofilm dispersion, possibly by modulating global regulatory systems that trigger bacterial evacuation responses under perceived adverse conditions.

Moreover, nutrient shifts and environmental physical conditions can also significantly trigger dispersion. Sudden influxes of preferable carbon sources induce biofilm cells to disperse actively in pursuit of nutrients, whereas critical nutrient depletion can likewise force cells to seek new habitats [72]. Changes in temperature or pH have similarly been observed to precede biofilm dispersion events in several microbial systems, likely mediated through sensor kinase systems that subsequently regulate c-di-GMP pathways. In extreme cases, biofilms experiencing severe acidification and metabolic waste accumulation can deliberately degrade portions of their matrix to enable escape [73]. Some bacteria express matrix-degrading enzymes explicitly during dispersion. *Aggregatibacter* spp. secrete dispersin B, a glucan hydrolase that solubilizes their own EPS during starvation-induced dispersion. Mutants deficient in this enzyme form overly stable and recalcitrant biofilms [74]. Similarly, *P. aeruginosa* upregulates alginate lyase and PelA hydrolases during late-stage biofilm maturation, loosening the matrix structure and facilitating dispersal [3]. Additionally, induction of prophage-mediated lysis within biofilms indirectly facilitates dispersal by creating voids and disruptions in the matrix, prompting neighboring cells to relocate [75].

Overall, dispersion is an active response that involves breaking down the EPS matrix and recovering motility. Understanding dispersion cues can support targeted interventions to control biofilm release and reduce contamination risks in food processing facilities.

### 4.2. Risks and Microbial Spread Associated with Dispersion

The dispersion of biofilms poses substantial food safety challenges due to the sudden release of previously matrix-protected microbial cells into the surrounding environment, significantly elevating contamination risks. A covert biofilm within food processing equipment, upon dispersion, can instantly release massive numbers of pathogenic bacteria into the product or processing environment. Simulation studies have illustrated scenarios where hidden *L. monocytogenes* biofilms within wet processing lines suddenly disperse, simultaneously releasing thousands of cells into product streams, leading to rapid, high-dose contamination within a single production day [76]. Such events explain intermittent yet severe contamination outbreaks in food manufacturing facilities, where pathogens typically undetected during routine monitoring suddenly appear in high concentrations following a dispersion event. Moreover, biofilm dispersion is inherently sporadic and unpredictable, complicating detection efforts and routine monitoring practices.

Dispersed biofilm cells frequently exhibit enhanced adaptability and stress tolerance compared to typical planktonic cells due to pre-acquired stress resistance phenotypes (e.g., acid tolerance, oxidative stress resistance) developed within biofilms. Studies have reported that dispersed *Listeria* cells from dairy equipment biofilms exhibit shorter lag phases and achieve higher population densities in milk compared to planktonically inoculated cells, indicating that biofilm-derived cells are better primed for colonization and proliferation in new environments.

Dispersion events can further exacerbate contamination through extended microbial distribution. Detached bacterial clusters and individual cells can contaminate current production batches directly or disseminate to distant areas through water splashes, aerosolization, or dust-mediated transfer. In humid processing environments, *Listeria* biofilms continuously release free cells into surrounding water films, facilitating cross-contamination to adjacent workstations or equipment via cleaning water splash [64]. Similarly, in dry environments, biofilm fragments incorporated into dust particles can disseminate microbes to previously uncontaminated raw materials or packaging zones [77].

Moreover, biofilm dispersion can facilitate the spread of microbial resistance traits and genetic elements. Biofilms are recognized as hotspots for horizontal gene transfer (HGT), where dense microbial communities and abundant eDNA enhance plasmid, integron, and resistance gene exchanges among bacteria. Research indicates that rates of antibiotic resistance gene transfer via conjugation, transformation, and phage-mediated transduction within biofilms exceed those observed in planktonic cultures by several orders of magnitude [78]. Consequently, dispersion events not only distribute individual bacteria but also propagate antimicrobial and disinfectant resistance traits acquired within biofilms. This phenomenon effectively broadens the scope of resistant microbial populations and complicates subsequent sanitation efforts and public health management. Thus, biofilm dispersion acts as a double-edged sword, essential for bacterial survival yet posing severe, latent contamination threats in food safety contexts. Awareness and proactive monitoring of dispersion risks, integrated into routine sanitation practices, are therefore essential to prevent uncontrolled contamination events during food production.

### 4.3. Intervention and Control Strategies for Biofilm Dispersion

Given the substantial risks associated with biofilm dispersion, the food industry must adopt proactive strategies to actively manage or induce controlled dispersion, minimizing potential hazards rather than passively awaiting spontaneous events. One strategic approach involves inducible controlled dispersion, where biofilms are deliberately disrupted under monitored conditions immediately followed by antimicrobial treatment. Low-dose NO-donor compounds can be introduced into processing equipment immediately prior to sanitization to trigger synchronized biofilm dispersion [79]. Subsequent sanitation protocols rapidly eliminate dispersed cells. Similarly, studies by Hentzer et al. [80] have demonstrated that incorporating halogenated furanone-based QSIs into cleaning regimens destabilizes *P. aeruginosa* biofilms, prompting substantial premature detachment, thus enabling timely bacterial removal during sanitation.

Another practical strategy involves intensified physical removal methods and containment measures. Suspected mature biofilms can be isolated during sanitation procedures, employing rigorous physical removal techniques, such as high-pressure water jets, ultrasonic cleaning, or steam treatment, to dissolve or detach robust EPS structures [53]. Mechanical scrubbing, while effective, requires careful management to prevent equipment surface damage or secondary contamination through aerosolization. Crucially, detached biofilm materials must be immediately flushed out and removed, potentially using fine-mesh filters installed within cleaning circuits to prevent dissemination.

Biological inhibitors and biocontrol agents also provide valuable tools during biofilm dispersion. Targeted application of bacteriophage preparations following dispersion events can selectively kill detached bacteria. *Listeria*-specific phage mixtures have demonstrated effectiveness, achieving approximately two-log reductions in viable bacterial counts within 24 h on equipment surfaces while visibly disrupting biofilm structure [81]. Commercial bacteriophage products approved for food industry use are currently applied on conveyor belts or equipment surfaces, effectively suppressing biofilm regrowth and intercepting dispersed cells.

For persistent cells, the “wake up-and-kill” strategy is a good approach [63]. Incorporating nutrient flushes or mild thermal stimuli during cleaning procedures thus facilitates removal of reactivated persisters by subsequent disinfectants. Future developments may even target molecular inhibition of persister formation itself, ensuring all cells remain susceptible during routine sanitation cycles.

In summary, biofilm dispersion control necessitates comprehensive preemptive measures. Food manufacturers are already integrating enzyme formulations, signaling molecules, and real-time biofilm monitoring sensors into routine cleaning protocols, ensuring timely detection and intervention. Customized combinations of chemical signaling induction, biological inhibitors, and intensified physical removal methodologies promise significant reductions in biofilm dispersion risks, effectively preventing “Pandora’s box”-type contamination incidents while ensuring sustainable food production operations. Additionally, controlling biofilm dispersion requires not only chemical and physical interventions but also anticipatory monitoring and rapid-response systems.

## 5. Future Directions and Emerging Technologies

Emerging technologies are shifting biofilm control toward predictive, automated, and self-regulating systems. As summarized in Table 1, smart surfaces, biosensors, predictive analytics, and automated sanitation platforms represent the key innovations shaping this transition.

### 5.1. Data Integration and Predictive Modeling

Modern food sanitation is evolving to harness large datasets from processing lines and environmental sensors, enabling predictive models that assess biofilm risk in real time [82]. By integrating parameters like surface conditions, microbial counts, and cleaning schedules, such models can forecast where and when biofilms are likely to develop. For example, recent work developed mathematical models to simulate *Cronobacter sakazakii* (*C. sakazakii*) and *Enterobacter* biofilm formation on various food-contact surfaces under different temperatures and initial loads [83]. These models accurately predicted biofilm accumulation over time and helped identify safe operational limits (e.g., showing that daily refrigeration-unit cleaning is needed to prevent significant buildup) [83]. On the factory floor, data-driven risk modeling is being coupled with sensor feedback. Multivariate algorithms can analyze signals from flow meters, turbidity probes, and microbial sensors to detect early biofilm attachment and trigger interventions. One study demonstrated that a multi-sensor machine-learning system could monitor cleaning-in-place (CIP) efficacy in real time, using ultrasonic and optical fouling sensors to decide when equipment was clean enough to halt cleaning [84]. Such an adaptive approach prevents both under- and over-cleaning [84]. In essence, integrating sensor networks with predictive analytics allows a shift from reactive to proactive biofilm control. As these “smart sanitation” systems mature, they are expected to minimize biofilm-related downtime by anticipating contamination and optimizing cleaning cycles accordingly [83].

**Table 1:** Emerging intelligent and biotechnology-driven approaches for future biofilm control.

Intervention Strategies	Molecular Targets/Mechanism	Advantages	Limitations	Industry Applicability	References
Smart Antimicrobial Coatings (e.g., pH-responsive phage release)	Bacterial metabolism triggers antimicrobial release	On-demand action, extended protection	Material stability and food compatibility need validation	High-risk niches (drains, filler heads)	[85,86,87]
Real-time Biosensors (e.g., electrochemical, optical)	Detects early attachment and EPS changes	Early warning, real-time monitoring	High cost, requires calibration and maintenance	Smart food factories	[88,89,90]
CRISPR-based Antimicrobials	Precise cleavage of pathogen genes	High specificity, low resistance development	Not yet regulated, still in experimental stages	Future precision biocontrol	[91]

### 5.2. Smart Antimicrobial Surfaces and Responsive Coatings

Surface engineering offers another emerging avenue for biofilm control: creating coatings that not only resist microbial attachment but actively respond to the presence of bacteria. Nanomaterial-infused coatings are a foundation of this strategy. Metals like silver or copper in nanoform impart surfaces with continuous antimicrobial activity, disrupting cells before robust biofilms can form. A recent study [16] showed that a nanoscale *silica* coating on stainless steel significantly reduced *L. monocytogenes* adhesion and made cells easier to wash off during cleaning, without affecting food contact safety. Beyond static nanocoatings, researchers are designing “smart” coatings that release biocides or change their properties in response to microbial stimuli. One cutting-edge example is a pH-responsive polymer film encapsulating bacteriophages. When bacterial metabolism locally acidifies the environment, the coating releases phages that infect and lyse the biofilm bacteria. Zuo et al. [85] demonstrated this concept by embedding phages in a chitosan-silica matrix that dissolves under acidic biofilm conditions. In tests, surfaces coated with these capsules had >90% less *Pseudomonas* biofilm biomass compared to uncoated surfaces, as the phages were autonomously released to attack the developing biofilm.

Similar responsive systems have been developed that react to bacterial enzymes or signaling molecules. Coatings incorporating peptide antibiotics linked via enzyme-cleavable bonds, which are broken by microbial proteases to trigger drug release [86]. By remaining inert until bacteria are present, these self-defensive surfaces achieve on-demand antimicrobial activity, minimizing continuous chemical exposure and slowing resistance development [87]. Such smart antimicrobial coatings, ranging from light-activatable nanoparticles to enzyme-sensitive polymers, represent a promising strategy to passively keep equipment free of biofilms. As durability and food-compatibility of these materials improve, the food industry could apply them to high-risk niches (drains, filler heads, conveyor belts), where they would lie in wait to neutralize bacteria at the earliest stage of attachment.

### 5.3. Biosensors and Monitoring

Real-time monitoring technologies are being deployed to detect biofilms in situ before they become problematic. A variety of biosensor platforms, electrochemical, optical, and chip-based, now allow continuous surveillance of surface microbial activity in food processing environments. Electrochemical impedance sensors, for example, can non-destructively measure biofilm growth on equipment by detecting changes in an electrode’s impedance as cells attach and produce insulating EPS. Kumar et al. [88] recently reported an impedance-based sensor that quantifies biofilm volume on different materials in real time. They derived a calibration between impedance increase and biofilm biomass, enabling the sensor to estimate biofilm thickness on a surface within a multi-species biofilm system. Such impedance monitoring could be integrated into equipment (e.g., pipe walls or tank surfaces) to alert when biofilm accumulation reaches a threshold and sanitation is required. Optical methods are likewise advancing for food-related biofilm detection. One approach uses fiber-optic probes to detect the slight refractive index changes caused by initial bacterial attachment. In one study, a fiber-tip ball resonator sensor detected *P. aeruginosa* cells attaching at levels far below the threshold of visual detection, signaling the presence of an early biofilm before traditional assays could measure it [89]. There is also development of coatings that emit an optical signal upon binding specific microbial metabolites. A surface film might fluoresce when it contacts quorum-sensing molecules, providing a direct visual indicator of active biofilm formation. In parallel, wireless “Internet of Things” (IoT) sensors are emerging to integrate biofilm monitoring into facility-wide data systems. Shafaat et al. [90] demonstrated a chipless wireless sensor tag that can be affixed inside processing equipment; when biofilm bacteria grow on the tag’s conductive surface, they trigger a change in the tag’s electromagnetic signal that can be read remotely via near-field communication. This innovation allows completely non-invasive monitoring. The sensor broadcasts an alert through the IoT network once biofilm growth bridges the circuit on the tag. The integration of these biosensors with automated controls means that, in the near future, a factory’s cleaning system might receive immediate feedback from surfaces and dynamically adjust sanitation frequency or intensity.

Overall, real-time biosensors, whether measuring electrochemical signals, optical changes, or wireless resonance, provide the food industry with early warning systems to detect biofilm formation before it advances to resilient maturity, enabling a shift toward more timely and precise biofilm management.

### 5.4. CRISPR and Ecological Microbial Control

As an alternative to chemical disinfectants, biological strategies are being pursued to selectively target biofilms while sparing desirable flora. One high-tech approach adapts the CRISPR-Cas genome editing system as an antimicrobial tool. By programming CRISPR to recognize DNA sequences unique to a contaminant species, researchers can zero in on biofilm-forming pathogens with lethal precision. Engineered bacteriophages are a promising delivery vehicle for CRISPR antimicrobials: phages infect the target bacteria and introduce a CRISPR-Cas payload that cuts vital genes or plasmids, killing or disabling the cell from within. In a recent breakthrough, investigators created a four-phage cocktail armed with CRISPR-Cas systems that specifically kill *E. coli* in mixed communities. These CRISPR-enhanced phages were shown to penetrate and reduce *E. coli* biofilms, and importantly they prevented the emergence of phage-resistant mutants by simultaneously attacking the bacterial genome [91]. Such “phage-guided gene scissors” exemplify how CRISPR can be applied beyond editing, as a precision antimicrobial that causes negligible collateral impact on other microorganisms. Although not yet deployed in foods, this approach points toward future biofilm treatments that could, for example, selectively eradicate *Listeria* or *Salmonella* persisters lurking in a facility’s microbiome.

Meanwhile, other biological interventions leverage ecological competition to suppress biofilm pathogens. The use of benign microorganisms (probiotics or protective cultures) to exclude or disrupt undesired biofilm formers is gaining traction. In laboratory models, harmless bacteria introduced to surfaces can pre-emptively occupy attachment sites or produce antagonistic substances, thereby limiting pathogen biofilm development. Probiotic biofilm co-culture has shown particular promise against *L. monocytogenes*. In one study, stainless steel coupons were first coated with a biofilm of *Lactobacillus plantarum* (*L. plantarum*), a lactic acid bacterium, before exposure to *L. monocytogenes*. The presence of the *Lactobacillus* film did not eliminate *Listeria* attachment but made the pathogen’s biofilm significantly more susceptible to a sanitizer treatment, resulting in greater log-reductions than in *Listeria* biofilms alone [92]. This suggests that a background microbiota can “soften” pathogenic biofilms or inhibit their maturation. In real factory settings too, surveys have found that sites with more diverse environmental microbes sometimes have lower prevalence of *E. coli* or *Salmonella* biofilms, hinting that benign strains out-compete the pathogens [93]. Intentionally seeding surfaces with protective microbes (e.g., *Bacillus* spp. that produce anti-biofilm lipopeptides) are now being explored as a biocontrol strategy in food plants.

Complementing this, bacteriophage biocontrol is under active development. Phage mixtures can be applied as surface sanitizers that specifically infect and lyse target bacteria without chemical residues. Notably, phages can penetrate the EPS matrix and proliferate, effectively becoming “self-amplifying” treatments in a biofilm. Jin et al. [94] showed that a lytic phage (vB_SpuM_X5) could eradicate mature *Salmonella* biofilms on stainless steel by ≥1 log CFU reduction, even at relatively low phage doses, by enzymatically degrading the biofilm matrix and killing the embedded cells. Phage biocontrol has already seen practical trials (e.g., phage sprays for producing wash water), and for food-contact surfaces it could serve as a periodic treatment to knock down biofilm hotspots without harsh chemicals. The future of biofilm prevention in the food industry may lie in these precision biological tools.

CRISPR-based antimicrobials offer a gene-level, highly specific mode of attack against biofilm-forming bacteria, while ecological interventions such as beneficial microbes or bacteriophages aim to reshape surface communities toward safer microbiomes. However, despite their substantial promise, both CRISPR-based and phage-based interventions face practical barriers that currently limit their implementation in real food-processing environments. CRISPR antimicrobials require highly efficient delivery systems to reach target cells within dense biofilms, and concerns remain regarding off-target gene effects, environmental persistence, and regulatory approval. Similarly, phage-based treatments can suffer from host-range limitations, instability under fluctuating factory temperatures or pH, and the risk of bacterial resistance to phages. Additionally, consistent dosing, integration into existing CIP workflows, and compliance with food-safety regulations present further operational challenges. Integrating these emerging methods with conventional hygiene therefore requires careful optimization to overcome these practical constraints and ensure reliable, industry-scale biofilm control.

## 6. Conclusions

Biofilm eradication in food processing environments remains a formidable challenge. These matrix-encased microbial communities, protected by a robust extracellular matrix and harboring dormant persister cells, tolerate sanitizers and often evade detection, revealing the shortcomings of conventional sanitation methods. Effective eradication of biofilms in food processing environments requires stage-specific, mechanism-driven strategies targeting adhesion, EPS formation, maturation, and dispersion (Summary of intervention strategies based on different stages of biofilms was shown in Table 2). Looking forward, biofilm management will be increasingly shaped by smart technologies capable of early detection and automated decision-making. Real-time biosensors integrated into IoT monitoring networks will allow continuous tracking of microbial adhesion and trigger sanitation only when biofilms begin to form. Smart surfaces and responsive antimicrobial coatings may autonomously release bacteriophages or antimicrobial molecules when microbial activity is detected, reducing reliance on routine chemical cleaning. In parallel, predictive analytics and machine-learning models will enable factories to forecast high-risk contamination zones and dynamically schedule CIP operations. Together with automated sanitation systems and precision biocontrol tools such as probiotic coatings or CRISPR-enhanced phages, these innovations are expected to shift biofilm control from reactive cleaning toward proactive and intelligent prevention, establishing safer and more sustainable food production practices.

## Figures and Tables

**Table 2:** Summary of intervention strategies based on different stages of biofilms.

Biofilm Stage	Intervention Strategies	Molecular Targets/Mechanisms	Advantages	Limitations	Industrial Applicability	References
Initial Adhesion	Surface Engineering and Material Modification (e.g., nano-silica coating)	Alters surface hydrophobicity, charge, roughness	Non-biocidal, reduces bacterial attachment, easier cleaning	Limited coating durability, higher cost	Food-contact surfaces (stainless steel, conveyor belts)	[16]
	Natural Anti-adhesion Compounds (e.g., polyphenols, essential oils)	Alters bacterial surface hydrophobicity, blocks adhesins	Green, safe, food-compatible	Efficacy is concentration-dependent, volatile	Treatment of food processing equipment surfaces	[17,18,19]
	Competitive Exclusion by Probiotics	Occupies binding sites, secretes antimicrobials (e.g., bacteriocins)	Self-sustaining, no chemical residues	Requires robust, cleaning-tolerant strains	Dairy, meat processing equipment	[20]
EPS Formation and Maturation	Enzymatic Degradation (e.g., DNase, Dispersin B, proteases)	Degrades EPS components (eDNA, polysaccharides, proteins)	High specificity, enhances sanitizer penetration	Enzyme stability issues, high cost	Dairy industry, pipeline systems	[32,33,34,35]
	EPS-disrupting Surfactants (e.g., rhamnolipids)	Disrupts polysaccharide-protein interactions	Enhances antimicrobial penetration, biodegradable	Concentration-dependent, may affect product sensory	Food processing surfaces in combination with cleaners	[8,36,37]
	Microenvironment Manipulation (e.g., nutrient pulses, temperature, pH shifts)	Activates dormant cells, disrupts internal homeostasis	Increases antimicrobial susceptibility, no chemical residues	Difficult to control, requires precise operation	Liquid food processing systems	[48,49,50,51,52,53]
Maturation: Persisters and QS	“Wake-and-Kill” Strategy (e.g., mannitol, glucose)	Metabolic reactivation of persister cells	Effective against dormant cells, enhances eradication	Requires precise control of concentration and timing	High-risk areas (filler heads, pipelines)	[63,64]
	Quorum Sensing Inhibitors (QSIs, e.g., halogenated furanones, flavonoids)	Blocks QS signaling, inhibits EPS synthesis and persister formation	Low resistance risk, synergistic with sanitizers	Natural QSIs have low stability, synthetic ones are costly	Broadly applicable in various food processing environments	[40,41,65,66,67]
Dispersion	Induced Controlled Dispersion (e.g., NO-donors, QSIs)	Lowers c-di-GMP levels, activates dispersal mechanisms	Synchronized dispersal allows centralized removal	Timing is hard to control, risk of secondary contamination	Pipes, wet processing areas	[79,80]
	Physical Removal (e.g., high-pressure water, ultrasonication)	Mechanical disruption of EPS structure	Fast, effective, no chemical residues	May damage equipment, generates aerosols	Large equipment, hard-to-reach surfaces	[53]
	Bacteriophage Treatment	Specific lysis of dispersed cells	Self-amplifying, no residues, highly targeted	Narrow host range, potential for resistance development	Targeted pathogen control (e.g., *Listeria*)	[81,94]

## Data Availability

The original contributions presented in the study are included in the article, further inquiries can be directed to the corresponding authors.

## Abbreviations

The following abbreviations are used in this manuscript:

EPS	Extracellular polymeric substance
QS	Quorum sensing
eDNA	Extracellular DNA
c-di-GMP	cyclic-di-GMP
QSIs	Quorum sensing inhibitors
T4P	Type IV pili
PIA/PNAG	Poly-β-1,6-N-acetylglucosamine
SCVs	Small colony variants
PDEs	Phosphodiesterases
HGT	Horizontal gene transfer
CIP	Cleaning-in-place
IoT	Internet of Things
